# Supplementary material for: Dendrites endow artificial neural networks with accurate, robust and parameter-efficient learning
Source: ArXiv. 2024 Sep 13:arXiv:2404.03708v2. Preprint. [Version 2] (PMC11419189)
Supplement: Supplement 1 [file NIHPP2404.03708v2-supplement-1.pdf]

Supplementary Material

Supplementary Table 1. Minimum test loss scores obtained by each model on five benchmark datasets across various configurations and their corresponding test accuracy. Performance accuracy is listed as mean ± standard deviation across *N=5* initializations for each model.

	**MODEL PERFORMANCE – MINIMUM LOSS**				
**Models**	**MNIST**	**FMNIST**	**KMNIST**	**EMNIST**	**CIFAR10**
	**Test loss**				
dANN-R	0.0644±0.0013	0.3152±0.0093	0.4880±0.0044	0.6282±0.0068	1.3771±0.0067
dANN-LRF	0.0483±0.0018	**0.2871±0.0021**	**0.4342±0.0054**	0.5675±0.0070	1.2528±0.0141
dANN-GRF	**0.0471±0.0022**	0.2879±0.0040	0.4615±0.0133	0.5317±0.0034	1.2732±0.0065
dANN-F	0.0498±0.0020	0.2909±0.0067	0.4356±0.0104	**0.5210±0.0018**	**1.2133±0.0087**
vANN	0.0864±0.0089	0.3579±0.0072	0.6905±0.0255	0.6861±0.0101	1.5297±0.0256
	**Test accuracy (%)**				
dANN-R	98.090±0.0583	89.108±0.1955	87.260±0.1346	80.986±0.2621	52.044±0.0172
dANN-LRF	98.466±0.1058	**90.194±0.0543**	89.070±0.1144	82.638±0.2798	56.076±0.3200
dANN-GRF	**98.576±0.0809**	89.996±0.2132	88.460±0.1872	83.520±0.1262	55.478±0.2848
dANN-F	98.442±0.0765	89.984±0.1227	89.128±0.1979	**83.680±0.1944**	**58.176±0.4343**
vANN	97.858±0.2811	88.122±0.1492	**91.194±0.3330**	80.219±0.2291	46.680±0.8106

Supplementary Table 2. Characterization of probability density functions is shown in Figure 4**A**. Skewness, kurtosis, and range are calculated.

models	First hidden layer			Second hidden layer			Output layer		
	**kurtosis**	**skewness**	**range**	**kurtosis**	**skewness**	**range**	**kurtosis**	**skewness**	**range**
dANN-R	2.741	-0.308	2.961	0.088	-0.124	3.261	0.857	-0.753	3.092
dANN-LRF	2.157	-0.372	4.082	-0.103	0.074	4.540	1.192	-0.510	3.496
dANN-GRF	3.457	-0.545	4.700	0.638	0.001	4.173	1.393	-0.561	2.869
vANN	2.814	-0.234	2.019	2.059	0.090	1.373	1.905	-0.796	0.805

Supplementary Table 3. Test accuracy obtained by each model on five noise levels, i.e., increasing the standard deviation of the Gaussian noise, and their corresponding test loss against the FMNIST noisy dataset. Test accuracy and loss are listed as mean ± standard deviation across *N=5* initializations for each model.

	**MODEL PERFORMANCE – NOISY IMAGES**				
**Models**	**σ=0.0**	**σ=0.25**	**σ=0.5**	**σ=0.75**	**σ=1.0**
	**Test accuracy (%)**				
dANN-R	89.392±0.2071	83.388±0.2340	77.108±0.2064	70.206±0.3007	64.456±0.5200
dANN-LRF	89.310±0.0514	83.926±0.2560	**77.744±0.1853**	**71.73±0.1393**	**66.288±0.3290**
dANN-GRF	**89.616±0.3628**	83.718±0.1289	76.864±0.3725	70.490±0.4438	64.996±0.2247
dANN-F	**89.520±0.1814**	83.766±0.2763	77.392±0.4827	71.386±0.6403	65.942±0.1788
vANN	89.288±0.3654	**84.178±0.2922**	77.054±0.6884	70.624±0.5920	64.186±1.2748
	**Test loss**				
dANN-R	0.3167±0.0035	0.5896±0.0078	0.8985±0.0072	1.176±0.0221	1.3742±0.0206
dANN-LRF	0.2997±0.0018	**0.4519±0.0081**	**0.6224±0.0079**	**0.7826±0.0098**	**0.9251±0.0045**
dANN-GRF	**0.2932±0.0086**	0.4868±0.0113	0.7033±0.0159	0.8827±0.0202	1.0381±0.0185
dANN-F	0.2955±0.0038	0.4658±0.0111	0.6491±0.0156	0.8124±0.0119	0.9560±0.0086
vANN	0.404±0.0066	0.9869±0.0492	1.6652±0.0528	2.196±0.0583	2.6139±0.0827

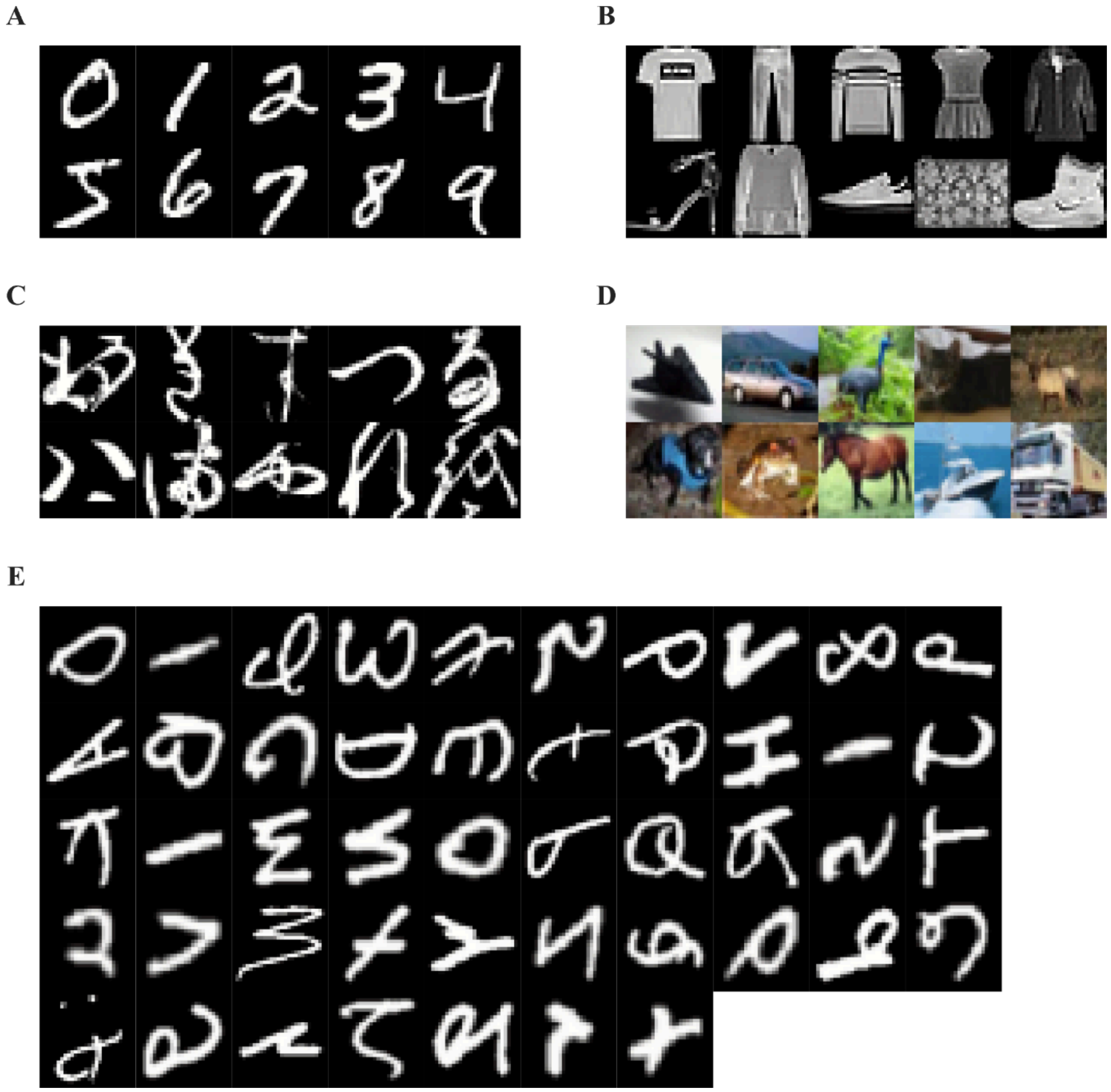

Supplementary Figure 1. The classification benchmark datasets. A. The MNIST dataset. **B.** The Fashion MNIST dataset. **C.** The Kuzushiji MNIST dataset. **D.** The CIFAR10 dataset. **E.** The EMNIST dataset.

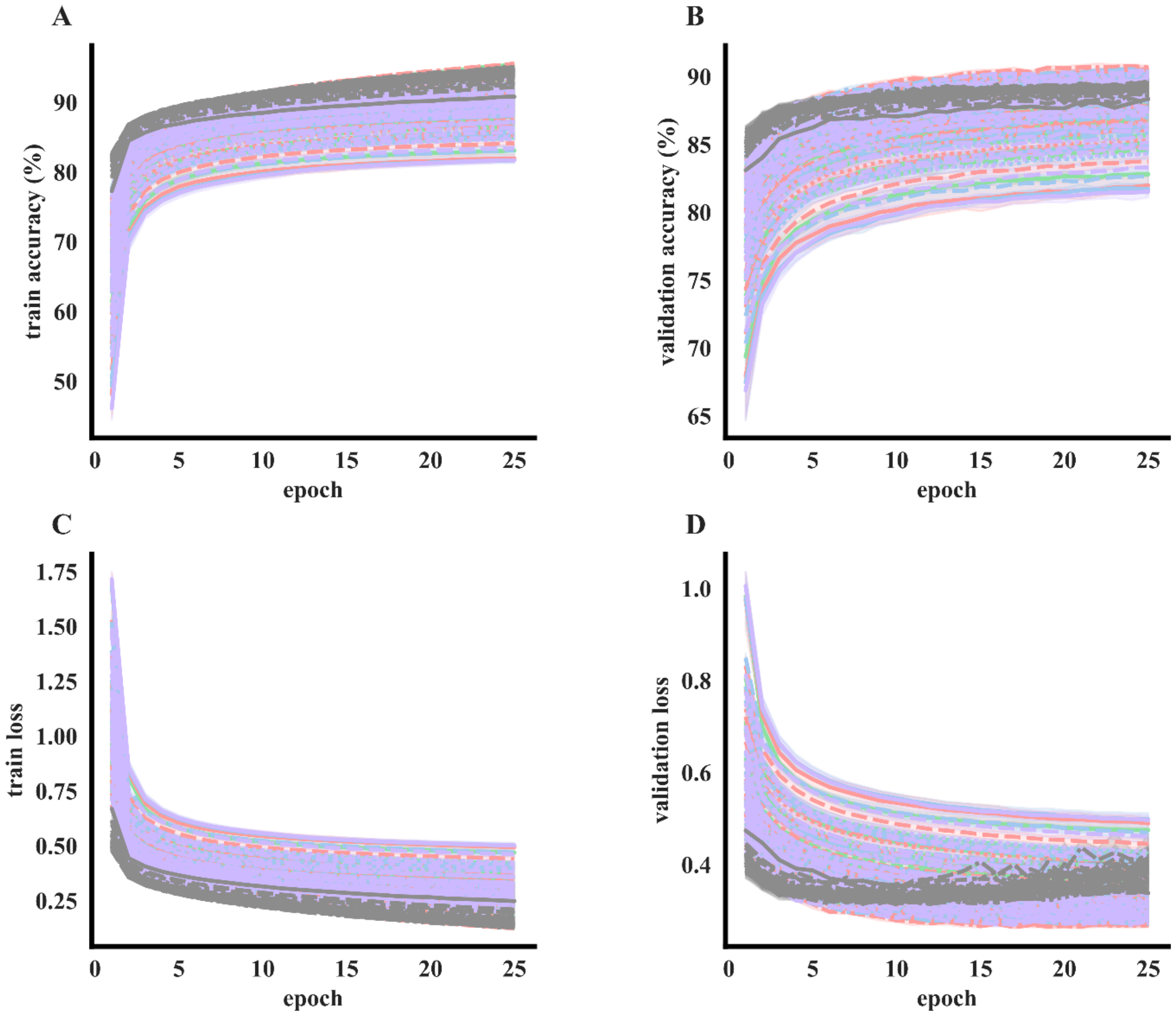

Supplementary Figure 2. Training and validation process of all models on FMNIST. A. Training accuracy for all model configurations (i.e., number of nodes in the hidden layers). **B.** Validation accuracy. **C.** Train loss. **D.** Validation loss. Shades denote standard deviation calculated across *N=5* initializations for each model.

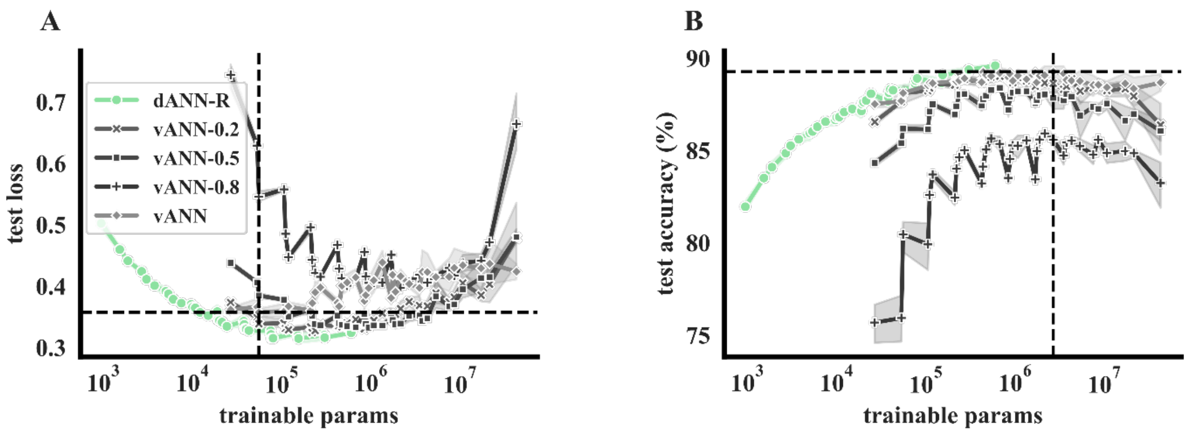

Supplementary Figure 3. dANNs vs vANNs with added dropout layers on FMNIST. A. Average test loss as a function of the trainable parameters of the models used: A dendritic ANN (dANN-R) with random inputs (green), and four variants of vANN (grey shades), with 0.2, 0.5, 0.8 dropout rates or no dropout layers, respectively. Horizontal and vertical dashed lines denote the minimum test loss of the vANN and its trainable parameters, respectively. The x-axis is shown in a logarithmic scale (log10). **B.** Similar to **A**, but depicting the test accuracy instead of the loss.

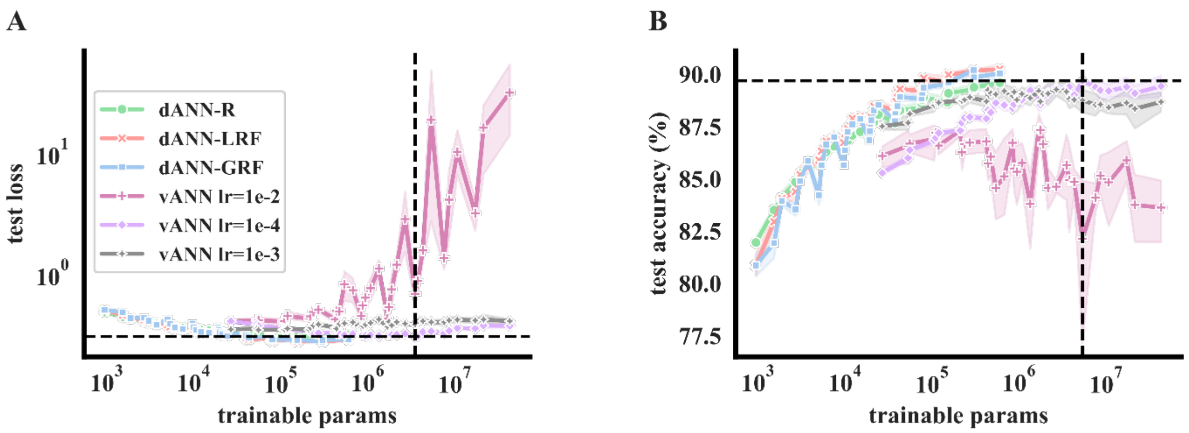

Supplementary Figure 4. Effect of learning rate on vANNs for the FMNIST dataset. A. Average test loss as a function of the number of trainable parameters of the six models used: Three dendritic ANNs with Random, LRF and GRF input sampling, and three vANN models: with 1e-2 (dark pink), 1e-3 (grey), and 1e-4 (light purple) learning rates, respectively. Horizontal and vertical dashed lines denote the minimum test loss of the control vANN and its trainable parameters, respectively. Both axes are shown in a logarithmic scale (log10). **B.** Similar to **A**, but depicting the maximum test accuracy instead of the loss. Note that the y-axis is shown in a linear scale here.

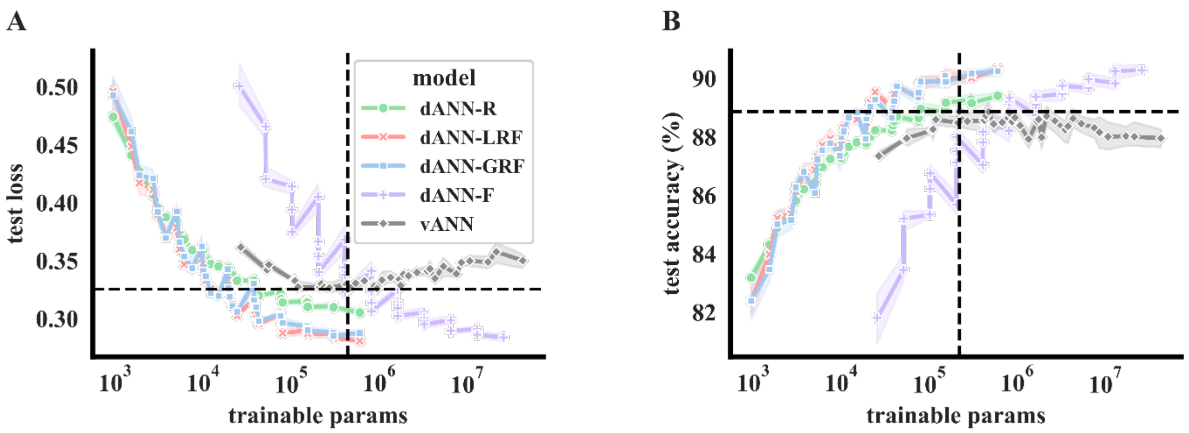

Supplementary Figure 5. Effect of early stopping on model performance on FMNIST. A. Average test loss as a function of the trainable parameters of the five models used: A dendritic ANN (dANN-R) with random inputs (green), a dANN with LRFs (red), a dANN with GRFs (blue), a dANN (dANN-F) with all-to-all inputs (orange), and the vANN model (grey). Horizontal and vertical dashed lines denote the minimum test loss of the vANN and its trainable parameters, respectively. The x-axis is shown in a logarithmic scale (log10). **B.** Similar to **A**, but depicting the test accuracy instead of the loss.

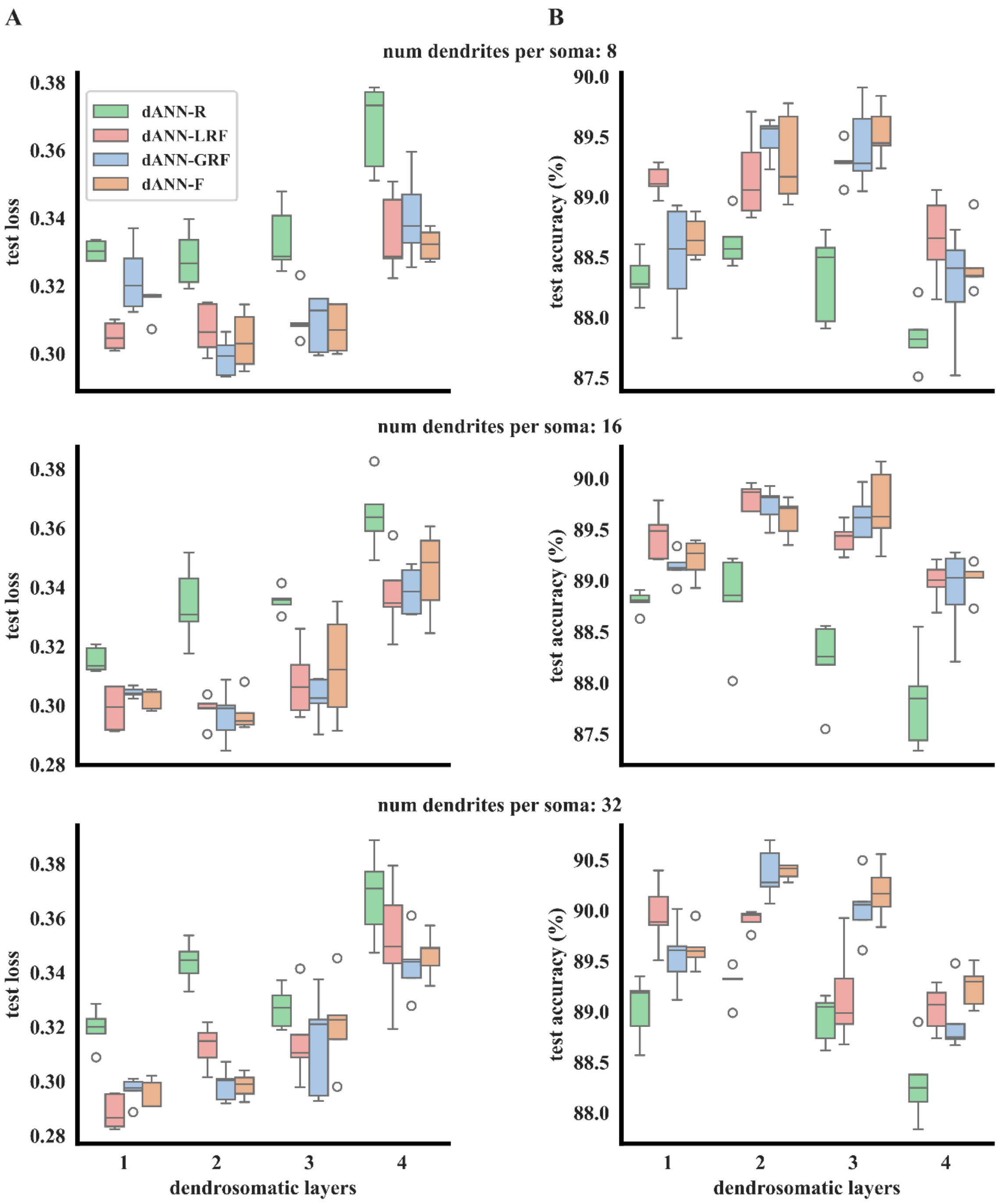

Supplementary Figure 6. dANNs trained on FMNIST can scale with depth. A. Test loss as a function of the number of dendrosomatic layers for the four classes of dANNs used: A dendritic ANN (dANN-R) with random input sampling (green), a dANN with LRFs (red), a dANN with GRFs (blue), and a dANN with all-to-all input sampling (orange). Horizontal and vertical dashed lines denote the minimum test loss of the vANN and its trainable parameters, respectively. The x-axis is shown in a logarithmic scale (log10). **B.** Similar to **A**, but depicting the test accuracy instead of the loss. Top to bottom: increasing number of dendrites per soma: 8, 16, 32, respectively. Number of somata was held constant to 256.

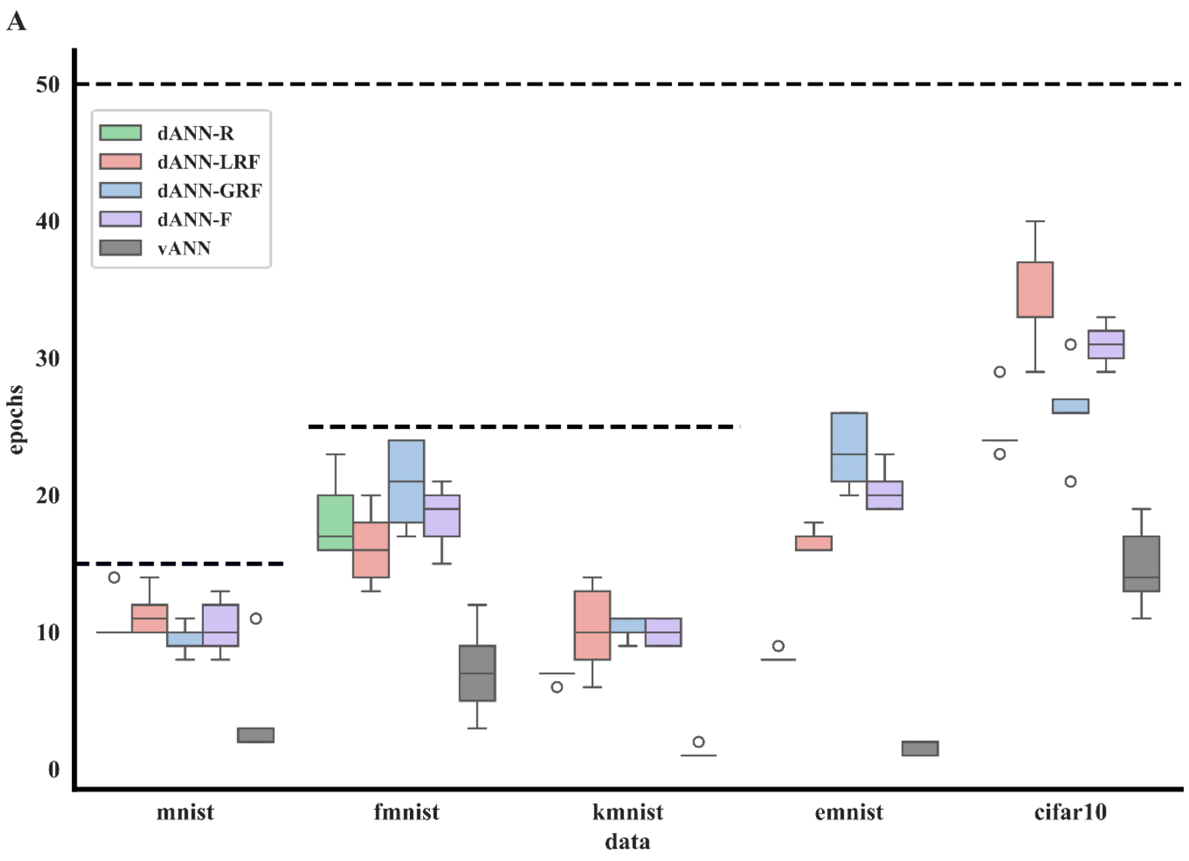

Supplementary Figure 7. Number of epochs needed to reach minimum validation loss. A. Number of epochs where validation loss was minimum for the dANN-R (green), dANN-LRF (red), dANN-GRF (blue), dANN-F (purple) and vANN (grey). Dashed lines indicate the number of epochs used for training: for MNIST, 15 epochs were used. For FMNIST and KMNIST, 25 epochs were used. For EMNIST and CIFAR10, 50 epochs were used.

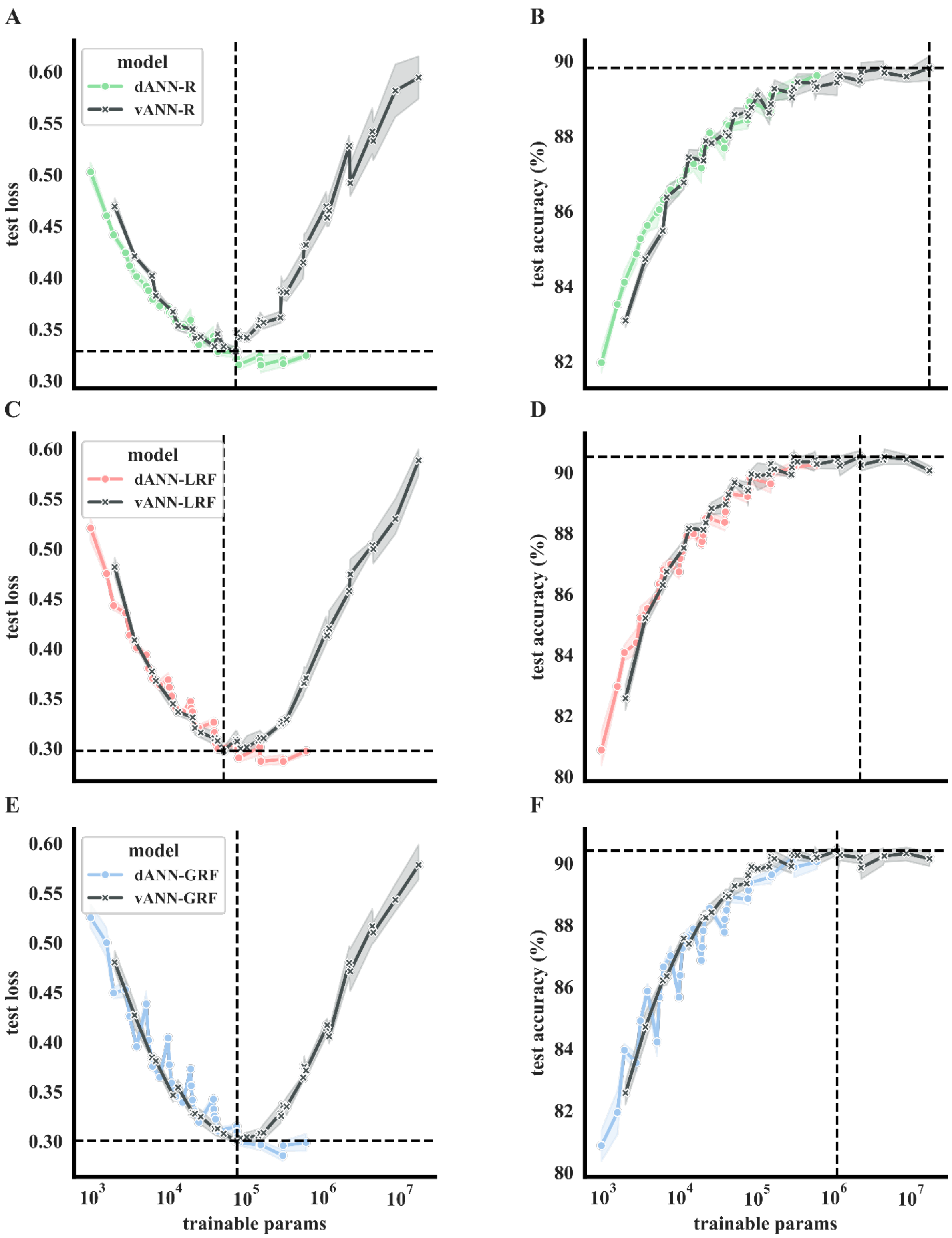

Supplementary Figure 8. Training and validation process of all models. A. Average test loss as a function of the trainable parameters of the dANN-R and vANN-R models. Horizontal and vertical dashed lines denote the minimum test loss of the vANN-R and its trainable parameters, respectively. The x-axis is shown in a logarithmic scale (log10). **B.** Similar to B, but depicting the test accuracy instead of the loss.

From top to bottom: random inputs, local receptive fields, global receptive fields. **C-D.** Similar to **A** and **B**, respectively, but depicting here the dANN-LRF and vANN-LRF. **E-F.** Similar to **A** and **B**, respectively, but depicting here the dANN-GRF and vANN-GRF.

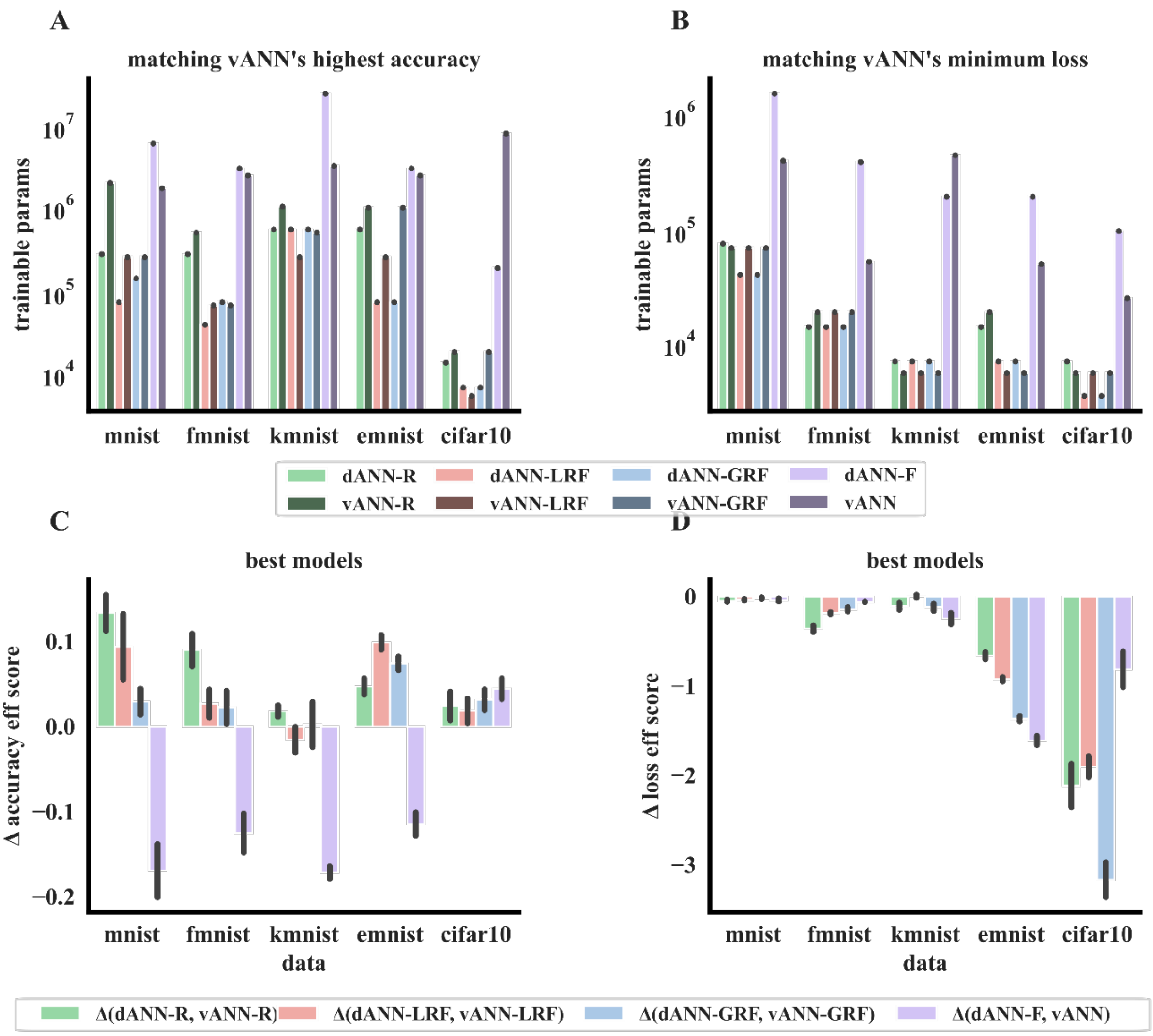

Supplementary Figure 9. dendritic vs. vANNs with sparse sampling. A. Number of trainable parameters that each model needs, dANN and vANN-R with random input sampling (light and dark green), dANN-LRF and vANN-LRF (light and dark red), dANN-GRF and vANN-GRF (light and dark blue), and dANN-F and vANN (light and dark purple) to match the highest test accuracy of the respective vANN. **B.** The same as in **A**, but showing the number of trainable parameters required to match the minimum test loss of the vANN. **C.** Difference (Δ) in accuracy efficiency score for dANN - vANN with all input sampling methods and all datasets tested. Test accuracy is normalized with the logarithm of trainable parameters times the number of epochs needed for convergence. The score is bounded in [0, 1]. **D.** Same as in **C**, but showing the difference (Δ) loss efficiency score. Again, we normalized the test score with the logarithm of the trainable parameters times the number of epochs needed for convergence. The score is bounded in [0, ∞). In all barplots the errorbars represent three standard deviations across N=5 initializations for each model.

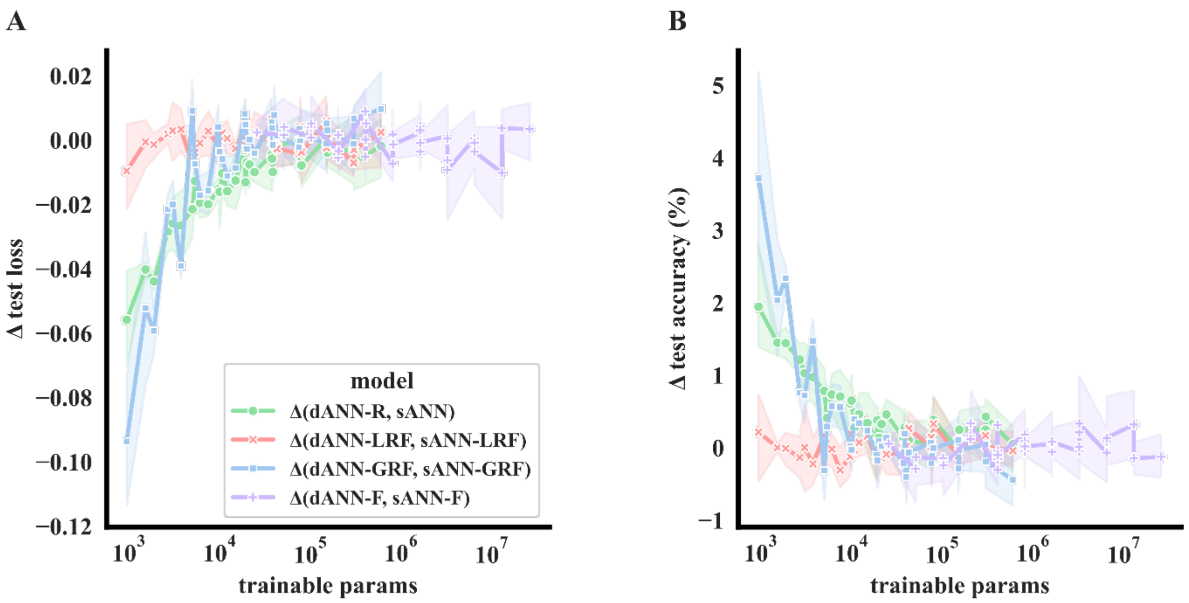

Supplementary Figure 10. Dendritic vs. random sparse ANNs. A. Average difference of test loss subtracting the sANN's loss from the dANN's one using models with the same input sampling method: random (green), LRF (red), GRF (blue), and all-to-all (orange). **B.** Similar to **A**, but depicting the difference in test accuracy instead of the test loss.

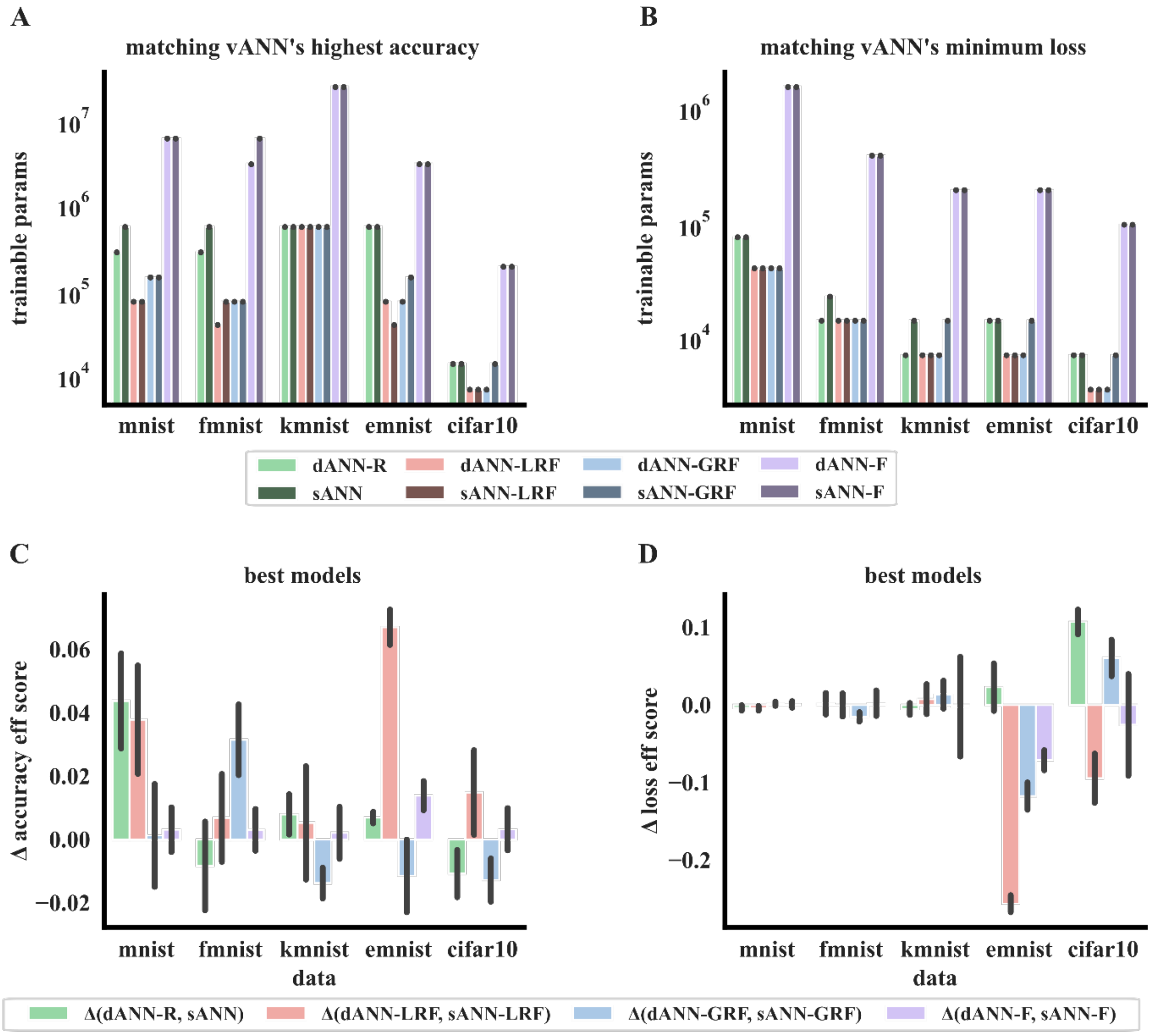

Supplementary Figure 11. dendritic vs. sparse ANNs. A. Number of trainable parameters that each model needs to match the highest accuracy of the vANN. Colors: dANN and sANN with random input sampling (light and dark green), dANN-LRF and sANN-LRF (light and dark red), dANN-GRF and sANN-GRF (light and dark blue), and dANN-F and sANN-F (light and dark purple) to match the highest test accuracy of the respective vANN. **B.** The same as in **A**, but showing the number of trainable parameters required to match the minimum test loss of the vANN. **C.** Difference (Δ) in accuracy efficiency score for the dANN – the sANN for all input sampling methods and all datasets tested. Test accuracy is normalized with the logarithm of trainable parameters times the number of epochs needed for convergence. The score is bounded in [0, 1]. **D.** Same as in **C**, but showing the difference (Δ) of the loss efficiency score. Again, we normalized the test score with the logarithm of the trainable parameters times the number of epochs

needed for convergence. The score is bounded in [0, ∞). In all barplots the errorbars represent three standard deviations across N=5 initializations for each model.
